# Protocol to drive human monocyte-to-macrophage polarization *in vitro* using tumor conditioned media

**DOI:** 10.1016/j.xpro.2022.101666

**Published:** 2022-09-19

**Authors:** Samantha George, Mirella Georgouli, Victoria Sanz-Moreno

**Affiliations:** 1 Barts Cancer Institute, Queen Mary University of London, John Vane Science Building, Charterhouse Square, London EC1M 6BQ, UK; 2 Oncology Experimental Medicine Unit, GlaxoSmithKline, Stevenage, SG1 2NY, UK

**Keywords:** Cell culture, Cell isolation, Cell-based assays, Cancer, Health sciences, Immunology, Cell differentiation

## Abstract

Tumor-associated macrophages (TAMs) are key contributors to antitumor immunity. Here, we present a protocol to drive human monocyte-macrophage differentiation using tumor-derived conditioned media, followed by phenotypic and functional characterization of TAMs *in vitro*. We describe CD14+ cell isolation from healthy human blood, and detail the procedure to induce macrophage polarization. Finally, we outline morphological assessment of macrophages, and validation of their functional behaviors with a tumor cell killing assay. This translatable-based approach can be applied to different cancer cell types.

For complete details on the use and execution of this protocol, please refer to Georgouli et al. (2019).

## Highlights

• Human translatable-based approach for investigating the role of the tumor secretome
• Using tumor cell-conditioned media to drive monocyte-to-macrophage polarization *in vitro*
• Morphological assessment of macrophages from brightfield microscopy images
• Assessing functional behaviors of macrophages with a tumor cell killing assay *in vitro*

## Abstract (editor highlights)

Publisher’s note: Undertaking any experimental protocol requires adherence to local institutional guidelines for laboratory safety and ethics.

## Graphical abstract

**Figure:**
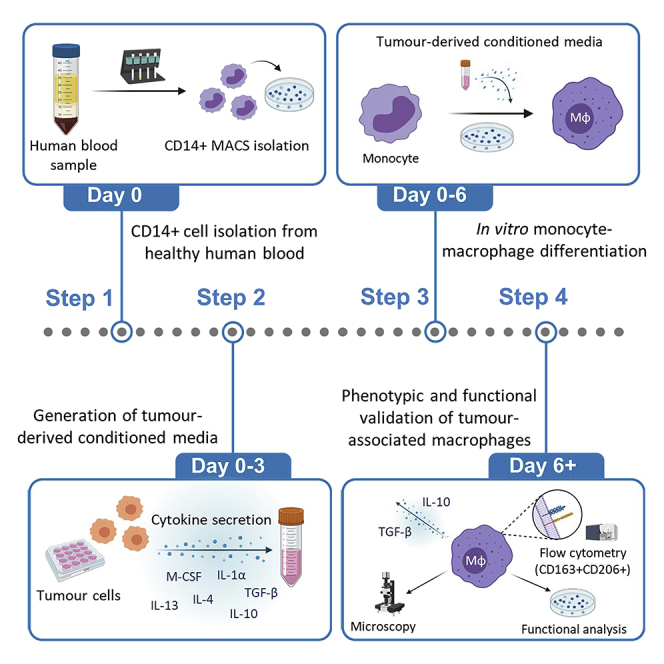

## Before you begin

### Institutional permissions

Study approval needs to be obtained for work with human biological samples prior to study start. This study was approved by the Guy’s Research Ethics Committee, study number 08/H0804/139, in addition to West London & GTAC Research Ethics Committee, study number 19/LO/1804. All experiments conformed to the relevant regulatory standards.

Transfer agreement will need to be set up between the research/academic institute and a suitable blood specimen provider, with the appropriate informed consent. Appropriate health and safety procedures should be followed, including institutional SOPs for handling and disposal of human blood samples using allocated sterile laminar flow hoods. In addition, users may require appropriate vaccinations to safely work with human biological samples, especially if samples are not screened for known human pathogens.

### PBMC isolation from human buffy coats

**Timing: (Day 0): 2 h approximately**

1. Peripheral blood mononuclear cells (PBMCs) from healthy donors are obtained from anonymized human buffy coats supplied by NHSBT (Tooting, London, UK).

***Note:*** Whole blood or blood cones can also be used.

2. Dilute 50 mL buffy coats 1:2 with PBS (−/− CaCl_2_/MgCl_2_; Gibco; room temperature).
  a. For example, 15 mL buffy coat and 30 mL PBS.
3. Using a standard 50 mL Falcon, gently layer 30 mL of diluted buffy coat on top of 15 mL Lymphoprep (STEMCELL Technologies; room temperature), by slowly pipetting to the side of the tube.

**CRITICAL:** Work quickly to maintain good cell viability. Layer the first few milliliters of diluted buffy coat very slowly onto the Lymphoprep layer, tilting the tube at a 45° angle, not to disrupt the interface.

4. Centrifuge in a swinging-bucket centrifuge at 800 × *g* for 30 min, at room temperature (15°C–23°C) with maximum acceleration and no brake.

***Note:*** PBMC isolation is performed via density gradient separation, which works on the basis that cells with different densities will be physically separated from one another. A common alternative for Lymphoprep is Ficoll-Paque.

**CRITICAL:** Use a swinging bucket rotor as opposed to a fixed-angle rotor, and make sure the brake deceleration is set to 0, to avoid disruption of the density gradient separation.

5. Collect PBMCs from the white interface layer using a disposable Pasteur pipette and transfer into a 50 mL Falcon tube containing fresh PBS (−/− CaCl_2_/MgCl_2_).
6. Top up solutions to 50 mL PBS and centrifuge at 200 × *g* for 10 min at room temperature with normal brake.

***Note:*** During washes, rather than aspirate the supernatant which can be harsh, simply pour out the supernatant slowly into a Virkon-filled container and dispose appropriately according to lab safety guidelines.

7. Discard the supernatant and repeat the washing step, resuspending in 50 mL PBS (−/− CaCl_2_/MgCl_2_).
8. Determine the number of live cells using a hemocytometer with Trypan Blue.
  a. 1:10 dilution often gives a good density for counting. Mix well before putting it into the chamber. Example: Mix 10 μL PBMC solution, 20 μL Trypan Blue, 70 μL PBS.

***Optional:*** This protocol continues without red blood cell (RBC) lysis buffer treatment, but some protocols incorporate this step depending on the isolation method used.

***Note:*** To help remove platelets after density gradient separation, PBMCs are centrifuged at 200 × *g* for initial washes.

9. Centrifuge sample at 300 × *g* for 10 min at room temperature with normal brake.

### Human CD14+ cell isolation

**Timing: (Day 0): 2 h approximately**

Human CD14+ cells are isolated from the PBMC population using magnetic-activated cells sorting (MACS) bead technology (Miltenyi), following the manufacturer’s instructions (https://www.miltenyibiotec.com/GB-en/products/cd14-microbeads-human.html#130-050-201). A sterile laminar flow hood is used. Please refer to manufacturer’s instructions for all the details and proposed optional steps.

10. Resuspend PBMCs in pre-cooled MACS buffer (PBS −/− CaCl_2_/MgCl_2_, 0.5% FBS, 2 mM EDTA) at a cell density of 10^7^ PBMCs/80 μL, mixed with 20 μL CD14 microbeads (Miltenyi). Mix cell solutions thoroughly and incubate for 15 min at 4°C.

***Note:*** Keep MACS buffer on ice throughout the process.

11. Add 1 mL of MACS buffer per 10^7^ PBMCs and centrifuge samples at 300 × *g* for 10 min at room temperature.
  a. For example, for 30 × 10^7^ PBMCs, top up with 30 mL MACS buffer.
12. After centrifugation, resuspend PBMCs in MACS buffer at a cell density of 10^8^ cells/mL.
13. Filter samples using a 70 μm cell strainer to homogenize the mixture and to reduce the risk of causing a blockage within the column.
14. Place the LS column in the Midi-MACS separator where a magnetic field is applied.
15. Pre-soak the LS column by adding 3 mL MACS buffer.

***Note:*** Take care at all steps to not introduce any air bubbles into the columns, pipetting carefully and never let the buffer run dry.

16. Apply the cell suspension (magnetically labeled cells with CD14 microbeads) onto the LS column, where unlabeled (CD14-) cell populations pass through the column and can be collected in a 15 mL Falcon tube.

***Note:*** If other cell populations are required for other experiments, retain this fraction for downstream isolation.

17. Perform washing steps by adding MACS buffer into the column (3 rounds of 3 mL washes). Only add new buffer when the reservoir is nearly empty.
18. Once all washing steps have been completed and the solution running through the columns appear clear, remove LS columns carefully from the Midi-MACS separator and place in a new 15 mL Falcon tube.
19. Add 5 mL of fresh MACS buffer onto the LS column and push the plunger down firmly to elute the CD14+ cell fraction which had been retained within the column due to CD14+ positive selection.

***Optional:*** To increase the purity of CD14+ cells, the eluted fraction can be enriched over a second LS column. Repeat the magnetic separation procedure by using a new column (steps 14–19).

20. CD14+ cells are counted and resuspended at 1 × 10^6^/mL in RPMI complete (10% FBS, 1% Penicillin-Streptomycin, 2 mM Glutamine), plated in the desired multiwell plate, and incubated at 37°C, 5% CO_2_.
  a. Standard tissue culture-treated multiwell plates are used for seeding monocytes (ultra-low attachment plates are not required).

### Determination of monocyte purity by flow cytometry

**Timing: 2–3 h approximately**

21. Take a portion of isolated CD14+ monocytes (<10^6^ cells) and keep on ice for the duration of the flow cytometry preparation.

**CRITICAL:** As staining is performed on live cells, keep samples on ice for the duration unless stated otherwise, and perform the antibody staining step at 4°C. This will maintain cell viability and prevent cell-surface receptor internalisation.

22. Centrifuge at 300 × *g* for 5 min at 4°C.
23. Wash once with FACS buffer (PBS−/−, 1% BSA, 2 mM EDTA, 0.1% NaN_3_) and centrifuge again at 300 × *g* for 5 min at 4°C.
24. Resuspend in 100 μL FACS buffer with 5 μL of FcR blocking solution (BioLegend) for 10 min at room temperature.

***Note:*** Keep a small proportion back for your unstained and DAPI single stained control.

25. Top up with FACS buffer and centrifuge at 300 × *g* for 5 min at 4°C.
26. Resuspend in solution of anti-human CD14 PerCP/Cyanine5.5 conjugated antibody in FACS buffer (1/20), for staining in a total volume of 100 μL. Vortex gently to mix.
27. Incubate for 30 min at 4°C in the dark.
28. Wash with FACS buffer and centrifuge at 300 × *g* for 5 min at 4°C.
29. Repeat wash step.
30. Resuspend samples in 300–500 μL DAPI solution (5 μg/mL in FACS buffer) for live/dead cell viability separation.
  a. For example, to make 5 mL of DAPI resuspension buffer, dilute 5 μL of 5 mg/mL DAPI stock in 5 mL FACS buffer.
31. In parallel, prepare compensation beads for compensation of fluorescence spill over from the fluorochrome-conjugated antibodies.
32. Add one drop of both ‘blank’ and ‘anti-mouse’ Igκ beads (Miltenyi) to 100 μL FACS buffer, and add the same dilution of CD14 antibody (5 μL), vortex gently. Top up to 300–500 μL FACS buffer.
33. Transfer solutions to FACS tubes and acquire data on a flow cytometer (BD FACS CANTO II).

***Note:*** Good CD14+ monocyte isolation leads to population purities of ≥95% CD14+ cells (See expected outcomes).

## Key resources table

**Table:** 

REAGENT or RESOURCE	SOURCE	IDENTIFIER
**Antibodies**		

CD14-PerCP/Cyanine5.5 (clone HCD14)	BioLegend (1:20)	325621 RRID: AB_893252
HLA-DR-FITC (clone L243)	eBioscience (1:50)	11-9952-41 RRID: AB_2572541
CD163-APC (clone eBioGHI/61 (GHI/61))	eBioscience (1:25)	17-1639-41 RRID: AB_2074540
CD206-PE (clone 15-2)	BioLegend (1:25)	321105 RRID: AB_571910
CD86-PE/Cyanine7 (clone IT2.2)	BioLegend (1:25)	305422 RRID: AB_2275754

**Biological samples**		

Human buffy coat	NHSBT Tooting	N/A - Anonymized healthy donors aged 17–65, male and female

**Chemicals, peptides, and recombinant proteins**		

Recombinant Human IL-4	PeproTech	200-04
Recombinant Human IL-10	PeproTech	200-10
Recombinant Human M-CSF	PeproTech	300-25
Recombinant Human IFN-γ	PeproTech	300-02
Lipopolysaccharides from Escherichia coli O111:B4 (LPS)	Sigma	L4391
4′,6-Diamidino-2-Phenylindole, Dilactate (DAPI)	BioLegend	422801
DMEM, high glucose, pyruvate	Gibco	41966029
RPMI 1640 Media	Gibco	21875034
Dulbecco’s phosphate buffered saline (PBS)	Gibco	14190094
PBS, calcium, magnesium	Gibco	14040133
Trypsin-EDTA (0.5%)	Gibco	15400054
Bovine serum albumin (BSA), fraction V	Roche	10735094001
Ethylenediaminetetraacetic acid disodium salt solution (EDTA)	Sigma	E7889
Sodium Azide (NaN_3_)	Sigma	S8032
Trypan Blue, 0.4% Solution	Lonza	17-942E
Fetal bovine serum (FBS; heat inactivated)	Gibco	10500064
Penicillin-Streptomycin	Gibco	15140122
Lymphoprep	STEMCELL Technologies	07801

**Experimental models: Cell lines**		

A375P	Prof. Richard Hynes HHMI, MIT, US	N/A
A375M2	Prof. Richard Hynes HHMI, MIT, US	N/A
WM88	Wistar Collection at Coriell Cell Repository	WC00123

**Software and algorithms**		

FlowJo	BD Life Sciences	https://www.flowjo.com/solutions/flowjo/downloads
ImageJ	(Schneider et al., 2012)	https://imagej.nih.gov/ij/download.html
GraphPad Prism	GraphPad Software, San Diego USA	https://www.graphpad.com/scientific-software/prism/

**Critical commercial assays**		

CFSE Cell Division Tracker Kit	BioLegend	423801
CD14+ Human microbeads	Miltenyi Biotec	130-050-201
LS columns	Miltenyi Biotec	130-042-401
MACS Comp Bead Kit, anti-mouse Igκ	Miltenyi Biotec	130-097-900
Human TruStain FcX	BioLegend	422302
Pierce™ BCA Protein Assay Kit	Thermo Fisher Scientific	23227
Amicon Ultra-4 Centrifugal Filter Units (3 kDa)	Millipore	UFC800324

**Other**		

70 μm cell strainer	Thermo Fisher Scientific	22363548
0.22 μm Stericup filtration system	Millipore	S2GPU02RE
Rely+On Virkon Tablets	LANXESS	N/A
Microscope	ZEISS	Axio Vert.A1
Microscope camera	ZEISS	Axiocam 202 mono
Cell culture CO_2_ incubator	BINDER	N/A
Swinging bucket centrifuge	Thermo Fisher Scientific	N/A
Microcentrifuge	Thermo Fisher Scientific	N/A
MACS multistand	Miltenyi Biotec	130-042-303
MidiMACS Separator	Miltenyi Biotec	130-042-302
BD FACS CANTO II flow cytometer	BD Biosciences	N/A
Microplate spectrophotometer	BMG LABTECH	N/A

## Materials and equipment

**Table:** FACS buffer

Component	Final concentration	Amount
PBS (−/−CaCl_2_/MgCl_2_)	N/A	443 mL
10% BSA	1%	50 mL
0.5 M EDTA	2 mM	2 mL
10% NaN_3_	0.1%	5 mL
**Total**	**N/A**	**500 mL**

Store refrigerated at 4°C, up to 4 weeks.

**Table:** MACS buffer

Component	Final concentration	Amount
PBS (−/−CaCl_2_/MgCl_2_)	N/A	495.5 mL
FBS	0.5%	2.5 mL
0.5 M EDTA	2 mM	2 mL
**Total**	**N/A**	**500 mL**

Store refrigerated at 4°C, up to 2 weeks if re-sterilised with a 0.22 μm filter.

**Table:** DMEM Complete culture media

Component	Final concentration	Amount
DMEM	N/A	445 mL
FBS	10%	50 mL
Penicillin-Streptomycin	1%	5 mL
**Total**	**N/A**	**500 mL**

Store refrigerated at 4°C, up to 4 weeks.

**Table:** RPMI Complete culture media

Component	Final concentration	Amount
RPMI	N/A	445 mL
FBS	10%	50 mL
Penicillin-Streptomycin	1%	5 mL
**Total**	**N/A**	**500 mL**

Store refrigerated at 4°C, up to 4 weeks.

## Step-by-step method details

### Part 1 - Macrophage differentiation using tumor-derived conditioned media

**Timing: 6 days**

This step utilizes tumor cell-derived conditioned media (CM) to induce *in vitro* macrophage differentiation and polarization. Published work has shown that manipulation of tumor cell intrinsic traits, such as blocking/knocking down myosin II activity, perturbs their ability to polarize macrophages to a pro-tumoral state via changes in secreted factors (Georgouli et al., 2019).

#### Day 0

1. Seed 1 × 10^6^ CD14+ monocytes in 1 mL of RPMI complete (10% FBS, 1% Penicillin-Streptomycin, 2 mM L-glutamine) in a 24-well plate and incubate at 37°C, 5% CO_2_.
  a. If you need a large cell number for functional assays downstream of monocyte-macrophage differentiation, you can scale up to these culture conditions:
    i. 2 × 10^6^ in a T12 well (2 mL culture media).
    ii. 4–5 × 10^6^ in a T6 well (4 mL culture media).

***Note:*** Primary cells are sensitive, so reduce cell plate handling outside of the cell incubator for too long.

2. To prepare tumor conditioned media, harvest melanoma cells from the cell culture flask. Aspirate culture media, wash with PBS (−/− CaCl_2_/MgCl_2_) and incubate with trypsin (Gibco) for 1–2 min at 37°C, 5% CO_2_.
3. Resuspend detached cells in DMEM complete (10% FBS, 1% Penicillin-Streptomycin), centrifuge at 300 × *g* for 5 min and resuspend in fresh DMEM complete.
4. Seed 2.5 × 10^5^ melanoma cells in 2 mL/T6 well of DMEM complete overnight. Plate replicate wells or adjust to your needs based on final protein yield required.
  a. If you need a large volume of conditioned media, you can scale up to these culture conditions:
    i. 1.5 × 10^6^ tumor cells in a 10 cm dish (10 mL culture media).

#### Day 1

5. Discard culture media from melanoma cell cultures. Wash tumor cells twice with PBS (+/+ CaCl_2_/MgCl_2_; Gibco).

***Note:*** The presence of Ca^2+^/Mg^2+^ promotes adhesion and helps prevent cells detaching during the wash steps.

6. Add 1 mL of serum-free DMEM (1% Penicillin-Streptomycin) per T6 well.
  a. If scaled up to a 10 cm dish, add 6 mL serum-free DMEM.
7. Incubate cells at 37°C, 10% CO_2_ for 48 h for media conditioning.

#### Day 3

8. For processing of conditioned media, harvest media from cell culture wells and centrifuge at 500 × *g* for 5 min to discard dead cells and debris.

**CRITICAL:** Make sure the conditioned media is kept on ice after harvest to maintain intact protein composition.

9. Transfer the supernatant into Amicon-Ultra 3 kDa filter-pore falcons (Millipore) and centrifuge at 4,000 rpm in a swinging bucket rotor for 45 min at 4°C.
  a. Protein solutions should reduce to volumes of 200–400 μL when concentrating 4 mL conditioned media, but will vary depending on starting volume.
10. Quantify conditioned media samples via BCA assay (Pierce) to calculate protein concentration levels as per manufacturer’s instructions (https://www.thermofisher.com/order/catalog/product/23227).
  a. Always titrate conditioned media with several dilutions to find an optimal concentration to analyze from the BCA standard curve (1:2-1:5 in PBS works well).

***Note:*** Run serum-free DMEM as your blank (same dilution factor to match your samples).

11. Normalize protein from each condition by resuspending desired conditioned media volumes into RPMI complete media at a concentration of 70 μg/mL (2×).
12. Carefully take out 50% media from macrophage T24-wells (500 μL) and add 500 μL of 2× protein solution, so you have a final solution of 35 μg/mL (1×).

***Note:*** Be careful to pipette slowly at this stage, to the side of the well, not to disrupt the monocytes. Replenishing media this way helps to reduce metabolic waste and increase viability.

13. For treatment, include a negative control of monocytes alone (RPMI media only). IL-4 (20 ng/mL), IL-10 (20 ng/mL), and M-CSF (50 ng/mL) can be used as positive controls for inducing alternatively-activated macrophages. IFN-γ (20 ng/mL) plus LPS (100 ng/mL) can be used to induce classically-activated macrophages.

***Note:*** IL-10 induces mainly CD163 expression and IL-4 induces mainly CD206 expression.

#### Day 6

14. Acquire bright-field images of macrophages at 10–20× magnification in order to analyze cell morphological differences by ImageJ software (see part 2 – Morphological assessment of macrophages).
15. Collect macrophage supernatants from all the conditions and transfer them into separate Eppendorfs.
16. Add 1 mL pre-cooled PBS (−/− CaCl_2_/MgCl_2_) to each well and place plates on ice for 30 min where cells will start to round up and detach.

***Optional:*** PBS can be supplemented with 2 mM EDTA to promote cell detachment.

17. In the meantime, spin down cell supernatants at 300 × *g* for 5 min to collect any floating cells, and resuspend in 100 μL FACS buffer (PBS−/−, 1% BSA, 2 mM EDTA, 0.1% NaN_3_).

***Note:*** Perform all centrifugation steps at 4°C. Floating cells will likely be monocytic or apoptotic.

18. After cells start to detach, gently scrape off the cells with the flat end of a P1000 tip, then with a new tip, pipette up and down before transferring cells into a fresh Eppendorf tube.
19. Combine cells with the supernatant fractions, centrifuge tubes at 300 × *g* for 5 min and perform a second wash with 1 mL FACS buffer.
20. Centrifuge at 300 × *g* for 5 min at 4°C, resuspend in 100 μL FACS buffer and human TruStain FcX blocking solution (1:20; BioLegend) and incubate for 10 min at room temperature. Vortex gently.

***Note:*** Before the next step, either take a small proportion from each individual condition (creating a pool sample), or already have allocated a condition for your unstained and DAPI single stain control. Transfer into a new Eppendorf tube.

21. Spin down samples at 300 × *g* for 5 min 4°C, and resuspend in FACS buffer containing a mixture of a 4-color antibody panel, with a staining volume total of 100 μL:

**Table:** 

Component	Volume (per sample)
HLA-DR-FITC	2 μL (1:50)
CD86-PE/Cyanine7	4 μL (1:25)
CD163-APC	4 μL (1:25)
CD206-PE	4 μL (1:25)
FACS buffer	86 μL

22. Vortex samples gently and incubate for 30 min at 4°C in the dark.
23. Wash twice with FACS buffer and finally resuspend in DAPI solution (5 μg/mL) for live/dead cell separation and acquire live samples on a flow cytometer (BD FACS CANTO II).

***Optional:*** It is also possible to fix cells in 1% formaldehyde (10 min room temperature incubation), centrifuge and resuspend in FACS buffer kept at 4°C if you wish to analyze later or the following day.

24. In parallel, prepare compensation beads to correct for fluorescence spill over from the fluorochrome-conjugated antibodies (single stain controls).
  a. Add one drop each of both MACS Comp Beads (anti-mouse Igκ; Miltenyi Biotec) and one drop of MACS Comp Beads (blank) to 100 μL FACS buffer.
  b. For each single stained control, the same antibody dilution is added.
    i. For example, for the CD86-PE/Cyanine7 compensation tube, 4 μL are to be added to compensation beads.

***Note:*** In all FACS experiments, unstained controls shall be used to identify forward/side scatter parameters of the cell population and to determine background fluorescence. Single stains were used to determine the laser voltage and to correct spectral overlap. Fluorescence minus one (FMOs), single stained controls and isotype controls can also be used to aid in gating strategies.

### Part 2 – Morphological assessment of macrophages

**Timing: ½ day**

Morphological assessment of differentiated macrophages can be analyzed from brightfield microscopy images taken at day 6.

25. Acquire at least one 10× image and three 20× images at different parts of the well.
26. Import images into ImageJ software.
27. Automatic cell shape descriptor measurements (such as roundness, circularity and area) can be generating by using the ‘freehand selection’ tool to manually draw around the outline of cells.
28. Cells can also be manually categorized into distinct subsets such as elongated/spindle or spread/fried egg.
29. These shape descriptors allow distinction of different morphological states and the level of spreading or differentiation on the plate (see expected outcomes).

***Note:*** 20× images are required to have better resolution of cell morphological protrusions and outlines.

### Part 3 – Macrophage tumor cell killing assay *in vitro*

**Timing: 3 days**

***Note:*** This step investigates the functional behaviour of macrophages with regards to their ability to elicit tumor-cell killing. The assay was adapted from Bracher et al. (2007) and was optimized according to experimental needs.

30. Trypsinize and count melanoma target cells (A375M2, WM88), and label with CFSE (BioLegend) using the CFSE cell division tracker following manufacturer’s instructions (https://www.biolegend.com/de-de/products/cfse-cell-division-tracker-kit-9396).
31. Centrifuge tumor cells at 300 × *g* for 5 min and resuspend at 10–100 × 10^6^ cells/mL in 5 μM CFSE working solution.
32. Incubate cells for 20 min at room temperature or 37°C protected from light.
33. Quench the staining by adding 5 times the original staining volume of cell culture media (RPMI) containing 10% FBS.
34. Wash CFSE-labeled melanoma cells and macrophages in complete RPMI before the co-culture.
35. Add targets (10^4^ melanoma cells) to macrophages (10^5^ macrophages) at a ratio of 1:10 (targets: effectors) in complete RPMI and incubate for 48 h in 48-well plates in duplicates.
36. Upon cell harvesting with trypsin, wash the cells with FACS buffer and resuspend in DAPI solution (5 μg/mL) for viability staining and immediately acquire on a flow cytometer (BD FACS CANTO II).
37. Discrimination of two cell populations could be obtained by flow cytometry analysis:
  a. CFSE+DAPI+ (dead tumor cells).
  b. CFSE+DAPI- (live tumor cells).

***Note:*** This cytotoxicity assay detects tumor cell killing broadly without specifying if it is from phagocytosis. For a more specific assay for macrophage phagocytosis, please refer to the zymosan phagocytosis assay in Georgouli et al. (2019).

## Expected outcomes

**Figure 1:**
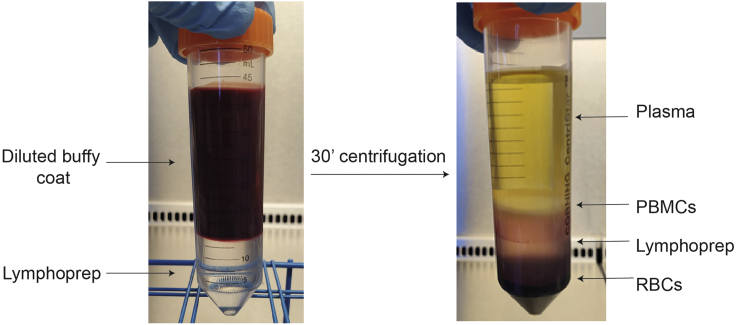
Density centrifugation of blood components into layers Diluted buffy coat samples are layered onto Lymphoprep and centrifuged at 800 × *g* for 30 min, with no brake. After density centrifugation, blood components should be separated into layers, purifying out a layer of peripheral blood mononuclear cells (PBMCs), from the plasma and red blood cells (RBCs). PBMCs are harvested for downstream monocyte isolation.

After density centrifugation, blood layers should be separated into distinct layers (Figure 1). The white layer interface will contain the PBMCs, which is extracted for downstream monocyte isolation.

**Figure 2:**
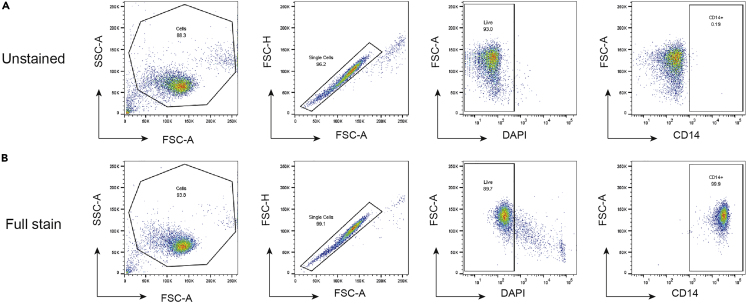
Flow cytometry gating strategy for purified CD14+ populations from human PBMC populations (A and B) Flow cytometry plots from unstained cells (A) and fully stained cells with DAPI and CD14 (B). From left to right, cells are first gated out from debris (FSC-A vs. SSC-A). Doublet discrimination is then performed to obtain single-cells only (FSC-A vs FSC-H; FSC-A vs. FSC-W). Viable cells are then gated as DAPI- (DAPI vs. FSC-A). Live cells are finally gated for CD14+ purity (CD14 vs. FSC-A).

After CD14+ monocyte isolation from PBMCs, flow cytometry is used to verify the population purity by analysis of CD14+ staining. Good purity of CD14+ cells is usually over 95% when gating for single live CD14+ cells (Figure 2).

**Figure 3:**
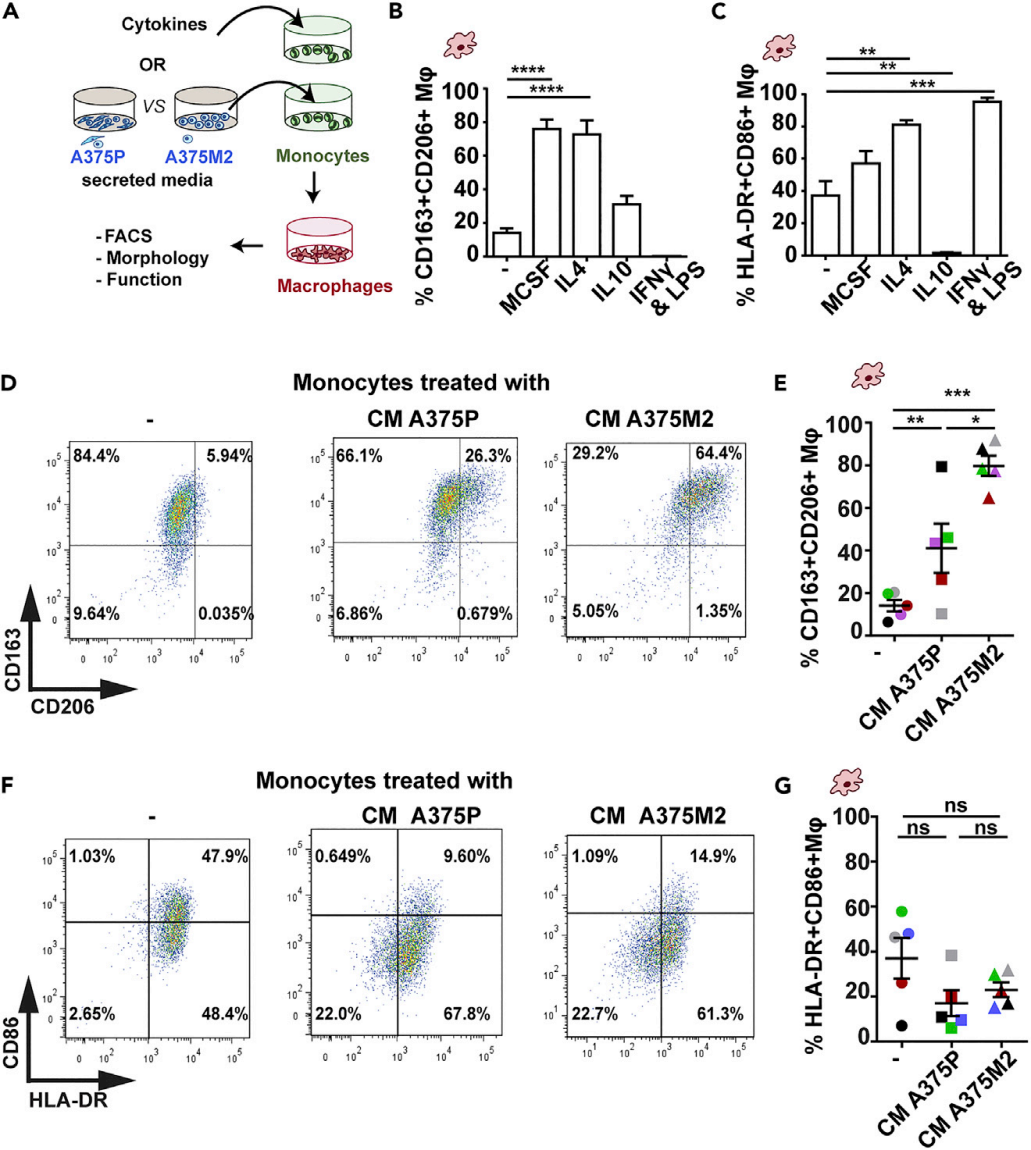
Polarization of CD163+CD206+ tumor-associated macrophages Figure adapted from Georgouli et al. (2019). (A) Schematic of treatment pipeline using tumor cell-derived conditioned media (CM). (B and C) Polarization of macrophages *in vitro* using cytokine controls, assessed by flow cytometry, showing % CD163+CD206+ (left), and % HLA-DR-CD86+ (right). (D and E) (D) Flow cytometry plots of CM-treated macrophages, showing the acquisition of cell surface markers CD163+CD206+, quantified in (E). (F and G) (F) Flow cytometry plots of CM-treated macrophages, showing the acquisition of cell surface markers HLA-DR+CD86+, quantified in (G).

After 6 days in culture, monocyte-derived macrophages will begin to demonstrate changes in phenotypic markers, alongside changes in morphology and function. FACS will determine the extent of cell-surface marker expression, such as the acquisition of alternative-activation markers CD163+CD206+ (Figure 3). The key observation here is that more invasive A375M2 tumor cells have a higher capacity to induce these surface markers, when compared to their less invasive counterpart A375P, due to differences in their secretome.

**Figure 4:**
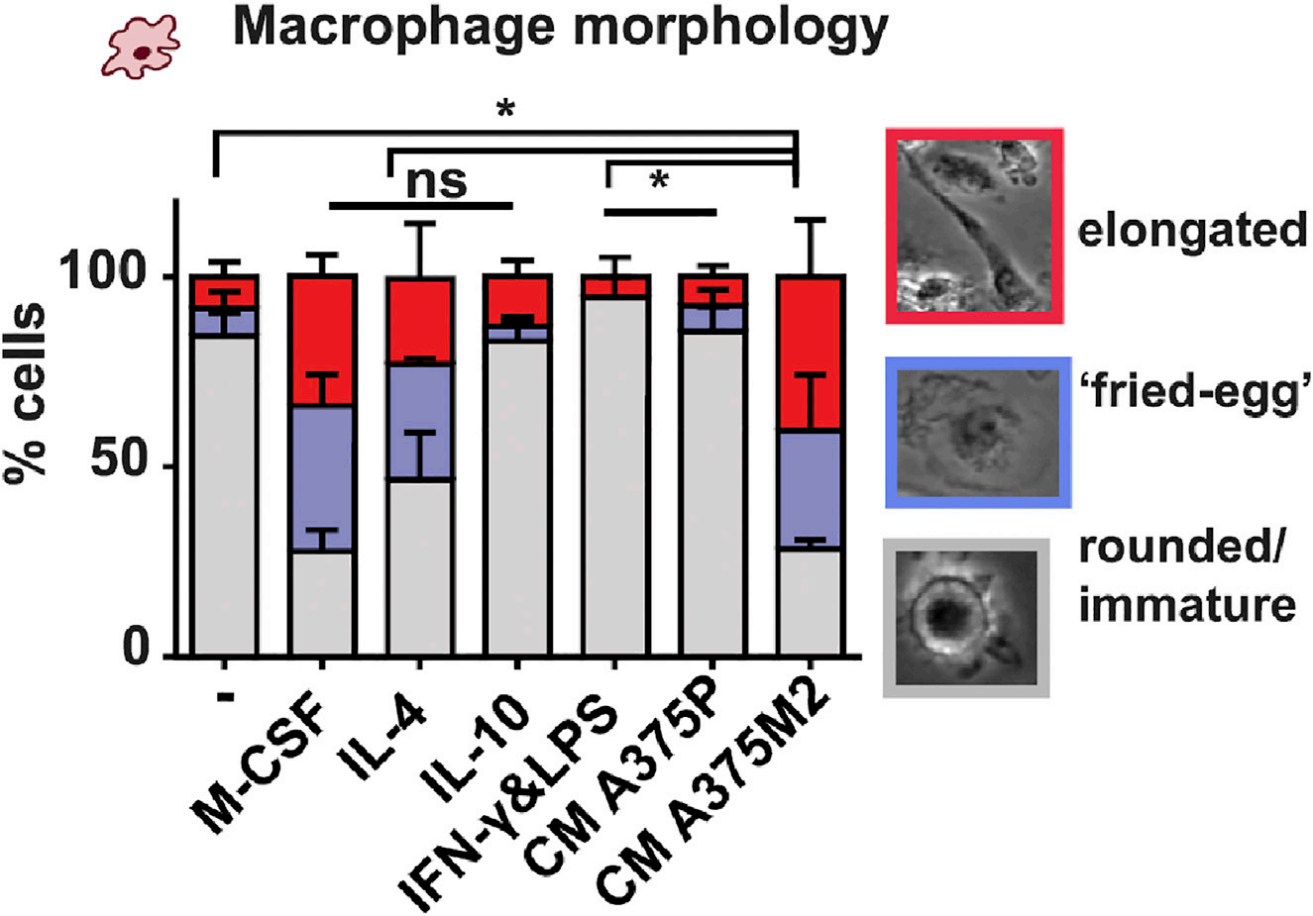
Assessment of macrophage morphology Figure adapted from Georgouli et al. (2019). Assessment of macrophage morphology from brightfield images, separating into 3 morphological categories of ‘elongated’, ‘fried-egg’ or ‘rounded/immature’. Treatments vary from RPMI baseline control ( - ), cytokine controls and treatment with tumor cell-derived conditioned media (CM).

Macrophage morphology can be assessed using brightfield images. Morphological data is portrayed in a stacked bar chart to highlight different phenotypic subsets (Figure 4). Macrophage attachment and spreading increases through the differentiation process, so morphological assessment can complement phenotypic characterization.

**Figure 5:**
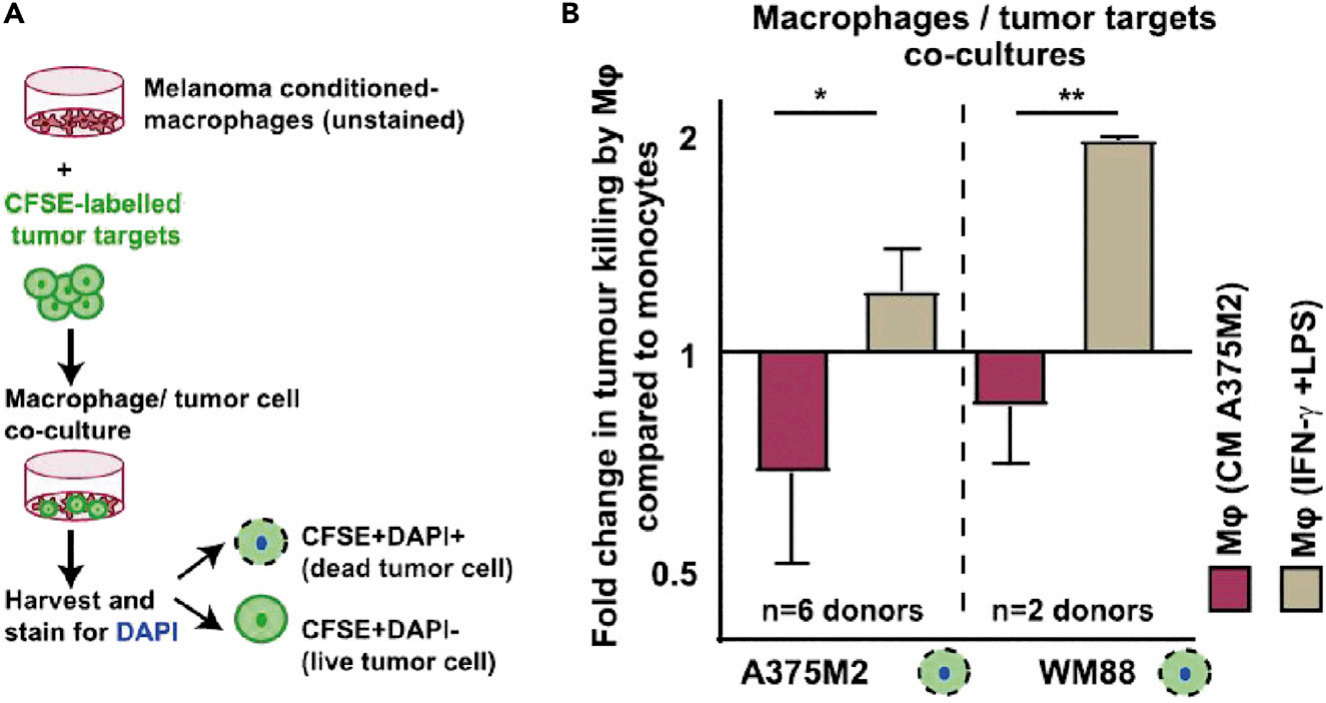
Tumor killing co-culture assay Figure adapted from Georgouli et al. (2019). (A) Schematic of CFSE labeling of tumor cells and flow cytometry pipeline. (B) Figure shows fold change of dead tumor cells (A375M2 and WM88) in co-cultures with macrophages treated with A375M2 conditioned media (CM), compared to co-culture with IFN-γ and LPS- treated macrophages.

When analyzing data from the cytotoxicity assay, fold changes of live:dead tumor cells can be calculated within different treatment groups, determining how tumor-associated macrophages can prevent tumor killing in *in vitro* co-culture (Figure 5). In this assay we observe that CM A375M2-treated macrophages, have a lower tumor killing capacity than macrophages treated with IFN-γ + LPS.

## Quantification and statistical analysis

Data from ≥3 donors from independent experiments are used to calculate statistical significance. Sample populations are tested for their normality. For phenotypical characterization, a one-way ANOVA with Tukey post-hoc test is performed. For classification of morphological differences from bright-field images, manual categorization is applied and analyzed by a one-way ANOVA with Bonferroni post-hoc test. ImageJ can also be used to generate automatic descriptors of cell shape, using the free-hand tool to draw around the cell perimeter. Cytotoxicity data is presented as fold change of dead tumor cells in co-culture with treated macrophages, versus with control untreated monocytes, where a t-test is performed.

## Limitations

This is an *in vitro* model which provides informative results under controlled conditions, but as these assays are performed without the full effect of the tumor microenvironment present, we advise that all observations are validated *in vivo* and by using patient-derived samples. There is significant inter-donor variability so experiments often need to be repeated more than 3 times (≥3 different donors). As this protocol utilizes primary human monocytes, care is taken to maintain good cell viability, as there is higher cell death in culture when comparing to immortalized cell lines. In addition, this protocol has been optimized with human melanoma cells, but could be applied to a range of different cancer cell types – it has been successfully used with pancreatic adenocarcinoma cell lines (data not shown).

## Troubleshooting

### Problem 1

Indistinct separation of layers during Lymphoprep gradient separation (before you begin: steps 1–5).

### Potential solution

Make sure to use a swinging bucket rotor centrifuge, and to disable the brake. Take care when layering diluted blood on top of Lymphoprep as to not mix the solutions, tilting the Falcon at a 45 degree angle. Make sure the Lymphoprep is a room temperature, and that the spin is also at room temperature.

### Problem 2

Low macrophage viability (steps 1–13).

### Potential solution

Make sure to work quickly when isolating and plating CD14+ from blood, using pre-cooled MACS buffer and to use fresh culture media when plating. Baseline M-CSF secretion of cancer cell lines can be assessed by ELISA. If this is very low or absent in conditioned media samples, supplementation with a very low level of M-CSF may be required. We have found, however, that monocytes grown in RPMI complete alone for 6 days have good cell viability, and that M-CSF further induced macrophages towards an alternatively-activated state, so we advise avoiding using this factor at high concentrations.

### Problem 3

Low monocyte yield (before you begin: steps 5; 10–20).

### Potential solution

A low yield of monocytes may arise from insufficient harvesting from the PBMC layer - make sure not to leave a large proportion of the white layer remaining. Be careful not to lose any cells during the wash steps, to spin for the full 10 min, and don’t dislodge/loosen the pellet when removing the supernatant. In addition, make sure you are adding sufficient CD14+ beads to your PBMC population and mix well before performing the magnetic separation. The yield of monocyte harvest is also highly dependent on the healthy donor-derived blood specimen, which may provide great variability in monocyte numbers.

### Problem 4

Low monocyte purity (before you begin: steps 10–20).

### Potential solution

Low monocyte purity could manifest as a low percentage of CD14+ cells when acquired on a flow cytometer. This can be caused by the excess use of CD14+ beads, which begin to bind un-specifically, or more commonly, through insufficient washing of cells while in the LS column separator. During these wash steps, do not add more wash buffer until the column is nearly empty, or alternatively you can increase the number of wash steps. In addition, make sure to filter cell suspensions to avoid column blockages, which could also cause a reduction in purity. Due to the high specificity of a positive selection, a RBC lysis step was not necessary with this protocol. However, this step may need to be considered depending on variations in PBMC source (primary blood material), donor-to-donor variability, processing/storage of sample, operator experience and PBMC isolation methods.

### Problem 5

Problems with flow cytometry compensation (steps 20–24).

### Potential solution

Ensure single stained controls are performed, at the same dilution of each antibody as in the experimental conditions, and to include an unstained control. Upon setting laser intensity, make sure each fluorophore is brightest in its own channel (voltage adjustment to correct fluorescence spillover). Antibodies should be titrated prior to use and ensure to adhere to flow cytometry tutorial/manual for compensation and sample acquisition.

### Problem 6

Do not see large differences of macrophage polarization when treated from different tumor cell line-derived conditioned media (steps 1–13).

### Potential solution

Make sure cells have a low passage number as those with high-passage number secrete suboptimal protein levels when compared with fresh low passage number tumor cells. Cell lines used in this study were used within passage range 1–6. It may also be advisable to titrate total protein levels when treating with tumor-derived conditioned media.

***Note:*** Conditioned media from melanoma cells is collected from cells grown in serum-free DMEM. More sensitive tumor cells where the absence of serum negatively affects their protein secretion or their viability, could be supplemented with low levels FBS (i.e., 1% FBS). Also note that phenol in DMEM will provide an absorbance within the BCA reading values. To circumvent this, make sure to add a blank of diluted serum-free DMEM as a control, and subtract this value before calculating final protein concentration of your conditioned media samples.

## Resource availability

### Lead contact

Further information and requests for resources and reagents should be directed to and will be fulfilled by the lead contact, Prof Victoria Sanz-Moreno (v.sanz-moreno@qmul.ac.uk).

### Materials availability

No materials were newly generated for this protocol. All materials mentioned above are commercially available.

## Data Availability

Data included with this protocol can be found in Georgouli et al. (2019).
